# Psychiatric Comorbidities in Patients with Psoriasis: A 10-Year Retrospective Epidemiological Study from a Tertiary University Center in Northeastern Romania

**DOI:** 10.3390/medicina61122190

**Published:** 2025-12-10

**Authors:** Bogdan M. Tarcau, Dan Vata, Ioana A. Popescu, Doinita Temelie Olinici, Adriana I. Patrascu, Angelica Postu, Ioana A. Halip, Madalina Mocanu, Marcel A. Gaina, Laura Gheuca Solovastru

**Affiliations:** 1 Dermatology Clinic, “St. Spiridon” County Emergency Clinical Hospital, 700115 Iasi, Romania; bogdan.tarcau@umfiasi.ro (B.M.T.); doinitzaganceanu@yahoo.com (D.T.O.); patrascuai@yahoo.com (A.I.P.); alinaioanahalip@gmail.com (I.A.H.); drmadalinamocanu@yahoo.com (M.M.); lsolovastru13@yahoo.com (L.G.S.); 2 Department of Dermatology, “Grigore T. Popa” University of Medicine and Pharmacy, 700115 Iasi, Romania; 3 Department of Morpho-Functional Sciences II, “Grigore T. Popa” University of Medicine and Pharmacy, 700115 Iasi, Romania; 4 Music Therapy Department, Berlin University of the Arts, 10623 Berlin, Germany; a.postu@udk-berlin.de; 5 Department of Psychiatry, “Grigore T. Popa” University of Medicine and Pharmacy, 700115 Iasi, Romania; marcel-alexandru_t_gaina@d.umfiasi.ro

**Keywords:** psoriasis, stress, depression, anxiety, quality of life

## Abstract

*Background and Objectives:* Psoriasis (PsO) is a chronic autoimmune inflammatory skin disorder frequently associated with psychiatric and psychological burden. The objective of this study was to determine the incidence of psychiatric comorbidities in patients with PsO from a dermatology clinic of a university hospital in northeastern Romania, thereby providing physicians with evidence to support more holistic and integrated care approaches. *Materials and Methods:* This is a 10-year retrospective epidemiological study on 2219 patients diagnosed with PsOwho were admitted to a Romanian university hospital between January 2013 and December 2022. Psychiatric and dermatological diagnoses were extracted from medical records using International Classification of Diseases—Tenth Revision (ICD-10) codes. Relative risks (RR) and 95% confidence intervals (CI) were calculated using Cox proportional hazards models, with *p* < 0.05 considered statistically significant. *Results:* Among 2219 patients, the most common psychiatric comorbidities were unspecified depressive disorder (120 patients, 5.4%, *p* < 0.01), mixed anxiety-depressive disorder (34 patients, 1.5%, *p* < 0.01), and mild depression (24 patients, 1.1%, *p* < 0.01). Female PsO patients demonstrated a relative risk (RR) [95% confidence interval (CI)] of 1.55 [1.12–2.14] (*p* = 0.007) for developing psychiatric comorbidities compared to males. Patients aged less than 18 years exhibited an RR [95% CI] of 2.032 [1.297–3.186] (*p* = 0.002) for psycho-emotional stress, the highest among all age groups. Notably, patients with concurrent psoriatic arthritis (PsA) showed a significantly elevated risk with RR [95% CI] of 1.546 [1.06–2.20] (*p* = 0.002). *Conclusions:* Depression and anxiety represent substantial psychiatric comorbidities in PsO patients, particularly affecting female individuals, younger patients, and those with concurrent PsA. These findings underscore the necessity for integrated mental health screening and psychological support within dermatological care protocols.

## 1. Introduction

Psoriasis (PsO) is a chronic autoimmune inflammatory skin disorder. The prevalence of PsO varies, according to the geographic location, from 0.91% to 8.5% [1]. In Romania, the estimated prevalence of PsO reaches 4.2% nationwide [2].

PsO has several clinical manifestations. The most common form of PsO, known as psoriasis vulgaris or chronic-plaque psoriasis, accounts for about 90% of all PsO forms [3]. It is characterized by salmon-pink, pruritic plaques covered by white-silvery scale, distributed symmetrically, mainly on the trunk, extensor surfaces of the extremities, and the scalp [3]. Besides PsO vulgaris, there are several other variants of PsO, such as guttate psoriasis, palmoplantar psoriasis, pustular psoriasis, inverse psoriasis, and nail psoriasis [3].

Guttate PsO is characterized by the acute onset of small, drop-like, erythematous plaques, usually following group-A streptococcal tonsilitis in children. One out of three children with guttate PsO will develop PsO vulgaris as they grow into adults [4,5]. Palmo-plantar PsO is characterized by hyperkeratotic, pustular plaques, frequently accompanied by painful and burning fissures, with significant functional disability [6]. Pustular PsO is characterized by multiple coalescing sterile pustules and erosions. It can be either localized or generalized, with an acute onset and diffuse background erythema [7]. Inverse PsO, also known as flexural PsO, is characterized by erythematous patches and fissures, localized in intertriginous areas, most often the axillary, anogenital, and inframammary folds [8]. Nail PsO affects up to 79% of patients with skin PsO and up to 80% of patients with psoriatic arthritis (PsA) and is characterized clinically by pitting, leukonychia, onycholysis, oil-drop spots, splinter hemorrhages, and nail bed hyperkeratosis [9].

PsO affects not only the skin and nails, but also other organ systems, proving that the underlying autoimmune inflammation is not skin-localized, but systemic [10]. Notably, elevated serum concentrations of several cytokines, namely TNFα, IFNγ, IL-6, IL-8, IL-12, IL-17A, and IL-18, are observed in comparison to healthy controls [11].

PsA affects about one-third of PsO patients, and because of its potential destructive progression has a great impact on the functionality and life quality of these patients [12,13,14]. It manifests clinically as monoarticular or poliarticular dactylitis and enthesitis. Nail PsO is a significant predictor of PsA [15].

The link between PsO and cardiovascular disease is well known. PsO patients have an increased risk of metabolic syndrome, ischemic heart disease, myocardial infarction, venous thromboembolism, and stroke [16,17,18,19]. Moreover, subclinical atherosclerosis is present even in mild forms of PsO and could be the first key element in developing the above-mentioned cardiovascular conditions [20].

Additionally, PsO patients are at an increased risk of developing Crohn’s disease, ulcerative colitis, diabetes mellitus, obesity, non-alcoholic fatty liver, and chronic kidney disease [21,22,23,24,25].

PsO patients frequently experience substantial psychosocial challenges, including but not limited to diminished self-esteem, body image concerns, and impaired quality of life [26]. Stressful life events have proven to be responsible not only for the onset but also for the exacerbation of PsO in a high proportion of patients, accounting for up to 70% of cases [27,28,29]. Depression does not appear to be linked to PsO severity, and even patients who experienced complete remission still suffered from depression [30,31]. Female PsO patients are more prone to develop depression [30,32]. In a large multicentric study on 3635 European dermatological patients diagnosed with PsO, non-melanoma skin cancer, skin infections, eczema, acne, atopic eczema, benign skin tumors, hand eczema, and leg ulcers, 10.1% of them suffered from depression in comparison to 4.3% of controls (Odds Ratio (OR) 2.40, 95% Confidence Interval [CI] 1.67–3.47) [33]. Moreover, the PsO subpopulation presented with an even higher risk, with an OR of 3.02, 95% [CI] 1.86–4.90 [33]. Clinical depression was reported in up to 19% of PsO patients based on the Diagnostic and Statistical Manual of Mental Disorders IV, in 12% of patients based on the International Classification of Diseases codes, while 28% reported depressive symptoms [34]. Female PsO patients are more prone to develop depressive symptoms or a depressive disorder in comparison to their male counterparts [35].

Furthermore, a large meta-analysis of 1,767,583 participants, of whom 330,207 had PsO, revealed that patients with PsO exhibited a higher chance of engaging in suicide attempts (OR 1.32; 95% CI = 1.14–1.54) and committing suicide (OR 1.20; 95% CI = 1.04–1.39) in comparison to individuals without PsO [36]. The authors of the meta-analysis also noted that the likelihood of experiencing suicidal tendencies was elevated among patients with more severe PsO and those of younger age [36]. Among dermatological patients, 12.7% reported suicidal ideation vs. 8.3% in controls (adjusted OR 1.24, 95% CI 0.95–1.62), but only PsO patients had a significant association (OR 1.94, 95% CI 1.33–2.82) [33]. Overall, 67.6% of the PsO subpopulation reporting suicidal ideation claimed that these thoughts were caused by the skin disorder itself [33].

PsO has also been associated with anxiety disorders, having a high odds ratio (OR 2.91, 95% CI 2.01–4.21), in a subpopulation of 17.2% dermatological patients vs. 11.1% in controls [33]. The prevalence of anxiety varies greatly, depending on the subtype: 15% (95% CI = 9–21) for social anxiety disorder, 11% (95% CI = 9–14) for generalized anxiety disorder, 9% (95% CI = 8–10) for unspecified anxiety disorder, and 5% for phobia anxiety (95% CI = 2–9) [37]. Anxiety symptoms alone are more frequent, with a prevalence of 34% (95% CI = 32–37) [37]. Mixed anxiety and depression disorder is common among psychiatric patients, but few studies on PsO have reported it. In a PsO population of 100, mixed anxiety depression disorder was reported in 8% of patients [32].

Moreover, schizophrenia has been associated with a higher risk for PsO, due to a genetic predisposition, and there are reports of such patients who received antipsychotic drugs and experienced remission, but also exacerbation of PsO [38]. Patients suffering from PsO had a pooled OR of 1.41 (95% CI = 1.19–1.66) for developing schizophrenia in comparison to non-psoriatic patients [39]. Patients with PsO have an increased risk of developing bipolar disorder, OR 2.33 (95% CI = 1.59–3.41), but the literature reports are scarce [40]. The main aim of this study was to determine the prevalence of psychiatric comorbidities in patients diagnosed with PsO in a dermatology clinic of a university hospital in northeastern Romania, in order to provide physicians, especially dermatologists, psychiatrists, and general practitioners, with a better understanding of the special needs of these patients. The second objective of the study was to evaluate the relative risk of different PsO subpopulations to develop psychiatric or mental health disorders. We hypothesized that specific demographic subgroups (younger age, female gender, and presence of PsA) would demonstrate elevated psychiatric comorbidity prevalence.

## 2. Materials and Methods

### 2.1. Study Design and Setting

All medical records of patients admitted to the Dermatology Clinic of “Saint Spiridon” Emergency Clinical County Hospital, Iasi, Romania, and diagnosed with PsO between 1 January 2013 and 31 December 2022 were systematically reviewed. This single-center, retrospective epidemiological study was conducted in accordance with the Declaration of Helsinki (2013 revision) and Good Clinical Practice (GCP) guidelines.

### 2.2. Data Extraction and Variables

Epidemiological data (age, gender, and living environment), personal medical history, and clinical diagnoses were extracted from 2219 medical records. The reported diagnoses were established on clinical criteria and further confirmed by laboratory results or skin biopsies when deemed necessary. Psychiatric diagnoses were extracted as recorded in the medical file using International Classification of Diseases—Tenth Revision (ICD-10) codes; the dataset did not distinguish between pre-existing psychiatric diagnoses and those identified during the hospital stay. Complete information regarding the temporal relationship between psychiatric and dermatological diagnoses was not available from the retrospective records.

### 2.3. Inclusion and Exclusion Criteria

Inclusion criteria: patients of any age, hospitalized in the Dermatology Clinic of “Saint Spiridon” Emergency Clinical County Hospital, Iasi, Romania, diagnosed with a form of skin or nail PsO between 1 January 2013 and 31 December 2022.

Exclusion criteria: patients who were not diagnosed with a form of skin or nail PsO.

### 2.4. Disease Severity Assessment

The PsO severity scores, which have been reviewed, include the Psoriasis Area and Severity Index (PASI), the Nail Psoriasis Severity Index (NAPSI), and the Psoriasis Scalp Severity Index (PSSI) [41]. PASI and PSSI range from 0 to 72, where 0 means no disease activity and 72 means maximal disease activity. PASI and PSSI scores between 0 and 4 are considered representative of mild disease, 5–10 moderate disease, and 11+ severe disease. NAPSI score ranges from 0 to 80 for just hands or feet and 160 for both, with a higher score representing a more severe nail involvement. The impact of PsO on life quality was assessed by the Dermatological Life Quality Index (DLQI) [42]. DLQI score ranges from 0 to 30 and is categorized as follows, according to the effect on the patient’s life: 0–1, no effect at all; 2–5, small effect; 6–10, moderate effect; 11–20, very large effect; and 21–30, extremely large effect. Notably, complete severity scores were not available for all patients, and analyses involving specific scales were limited to the subset with complete data (sample sizes specified for each analysis).

### 2.5. Statistical Analysis

The statistical analysis was conducted through the analysis of descriptive data expressed in the form of frequencies and percentages for categorical variables, and the mean ± standard deviation for numerical data. To determine the relationship between variables, bivariate correlation analysis was calculated using the Spearman correlation coefficient. Relative risks (RR) and the associated 95% Confidence Intervals (CI) were calculated based on the Cox proportional hazards model. A logistic regression model was used to predict a dependent data variable by analyzing the relationship between one or more existing independent variables. A *p*-value of less than 0.05 was considered to be statistically significant. All data analyses were carried out using the Windows 2.20 version of SPSS (IBM Corp., Armonk, NY, USA).

### 2.6. Ethical Considerations

This retrospective study utilized medical record data collected during routine clinical care. While initial data extraction included patient identifiers during the hospital documentation process, all variables used in this analysis were subsequently anonymized. According to Romanian institutional regulations and in accordance with Declaration of Helsinki principles for non-interventional research utilizing secondary data, formal ethics committee approval was obtained from the Ethics Committee of Grigore T. Popa University of Medicine and Pharmacy (Project identification code 293, approved 14 April 2023) prior to statistical analysis and manuscript preparation. Informed consent was obtained from all patients or their caregivers at the time of hospital admission for the use of their epidemiological and medical data in research studies. No information or photographs that could reveal patient identity were used in this analysis.

## 3. Results

### 3.1. Study Population Demographics

The demographics of the PsO study population (*n* = 2219) are presented in Table 1. The cohort included 1010 males (45.5%) and 1209 females (54.5%), with a mean age ± standard deviation of 52.46 ± 17.77 years. The majority (79.5%) were aged 40 years or older, with 42.6% aged 60 years or older. Urban residence was reported by 52.8% of participants, with 47.2% residing in rural settings.

### 3.2. Overall Psychiatric Comorbidity Prevalence

A total of 8% (177 out of 2219) of the investigated PsO patients suffer from at least one psychiatric comorbidity (*p* < 0.01).

The prevalence of specific psychiatric diagnoses, presented in descending order of frequency, included unspecified depressive disorder (120 patients, 5.4%), mixed anxiety-depressive disorder (34 patients, 1.5%), mild depression (24 patients, 1.1%), unspecified anxiety disorder (19 patients, 0.9%), moderate depression (11 patients, 0.5%), unspecified phobic anxiety disorder (7 patients, 0.3%), severe depression (3 patients, 0.1%), bipolar disorder (3 patients, 0.1%), paranoid schizophrenia (2 patients, 0.1%), schizoaffective disorder-depressive type (1 patient, 0.005%), and suicidal ideation (1 patient, 0.0005%). These findings are presented in Table 2.

### 3.3. Demographic Risk Factors for Psychiatric Comorbidities

Female PsO patients demonstrated significantly elevated relative risk for developing psychiatric comorbidities (RR [95% CI] = 1.55 [1.12–2.14], *p* = 0.007) compared to male patients. Specifically, female patients exhibited elevated risk for unspecified depressive disorder (RR [95% CI] = 1.87 [1.28–2.73], *p* < 0.01), though no significant gender differences emerged for anxiety disorders (*p* > 0.05). These findings align with broader epidemiological evidence demonstrating higher psychiatric disorder prevalence in women within the general population.

PsO patients with psychiatric comorbidities have a mean age of 54.56 with a median of 56.00 and a standard deviation of 10.751, while those without psychiatric comorbidities have a mean age of 52.37 with a median of 56.00 and a standard deviation of 18.091 (Table 3). Age distribution was similar between patients with and without psychiatric comorbidities, indicating no age-related sampling bias.

Female PsO patients have a RR [95% CI] of 1.37 [0.93–2.01] of suffering from significant psycho-emotional stress, while men have a RR [95% CI] of 0.73 [0.50–1.07] (Table 4). PsO patients under the age of 18 have a RR of [95% CI] of 2.032 [1.297–3.186] of suffering from significant psycho-emotional stress, while those over 60 years old have a RR [95% CI] of 0.567 [0.394–0.816]. Living in an urban setting is linked with a RR [95% CI] of 1.305 [1.040–1.637] of significant psycho-emotional stress, while the rural setting has a RR [95% CI] of 0.658 [0.472–0.918].

Female PsO patients have a RR [95% CI] of 1.55 [1.12–2.14] of having at least one psychiatric comorbidity (*p* = 0.007) compared to the male patients (Table 5). Living in an urban area is associated with a lower risk of developing a psychiatric comorbidity RR [95% CI] = 0.51 [0.37–0.69] (*p* < 0.001). Patients with psoriatic arthritis have a higher risk of suffering from psychiatric comorbidities, with an RR [95% CI] of 1.546 [1.06–2.20] compared to the PsO patients without psoriatic arthritis (*p* < 0.01).

Female PsO patients are more prone to develop unspecified depressive disorder RR [95% CI] = 1.87 [1.28–2.73] (*p* < 0.01). Patients living in the rural area have a higher tendency to develop mixed anxiety-depressive disorder RR [95% CI] = 2.68 [1.29–5.59] (*p* = 0.008) and unspecified depressive disorder RR [95% CI] = 1.72 [1.21–2.46] (*p* = 0.002) (Table 6).

PsO vulgaris, guttate PsO, palmoplantar pustular PsO, generalized pustular PsO, and nail PsO in the psychiatric subpopulation are associated with RRs [95% CI] of 0.97 [0.62–1.51], 0.73 [0.39–1.39], 1.44 [0.87–2.37], 0.95 [0.39–2.31], and 1.28 [0.93–1.76], respectively, but without any statistical significance (Table 7).

The incidences of the psychiatric comorbidities in each type of PsO subpopulation is represented in Table 8 (*p* > 0.05).

In the PsO and psychiatric study subpopulation, 2 (0.1%) patients are of minor age—RR [CI 95%] = 0.15 [0.03–0.63], 10 (0.5%) are aged 18–39–RR [CI 95%] = 0.42 [0.22–0.79], 105 (4.7%) are aged 40–59 RR [CI 95%] = 1.60 [1.31–1.97], and 60 (2.7%) are aged 60+ RR [CI 95%] = 0.79 [0.61–1.03] (*p* < 0.05) (Table 9).

Psycho-emotional stress has been detected in 17 (10.6%) PsO patients under 18 years old—RR [95% CI] = 2.03 [1.22–3.36], in 19 (6.4%) patients aged 18–39—RR [95% CI] = 1.22 [0.77–1.95], 52 (6.4%) patients aged 40–59—RR [95% CI] = 1.21 [0.91–1.61], and 28 (3%) in patients aged 60+ RR [95% CI] = 0.56 [0.38–0.82] (Table 10).

Unspecified anxiety disorder has been detected in 2 (1.3%) of PsO patients under 18 years old—RR [95% CI] = 1.46 [0.36–5.88], in 0 patients aged 18–39, 13 (1.6%) patients aged 40–59—RR [95% CI] = 1.85 [1.10–3.21], and 4 (0.4%) in patients aged 60+ RR [95% CI] = 0.49 [0.18–1.32] (Table 10).

Unspecified depressive disorder has been diagnosed in no minor-age patients, in 7 (2.4%) patients aged 18–39—RR [95% CI] = 0.43 [0.20–0.92], in 76 (9.3%) patients aged 40–59—RR [95% CI] = 1.71 [1.35–2.17], and in 37 (3.9%) patients aged over 60—RR [95% CI] = 0.72 [0.52–1.00] (Table 10).

### 3.4. Psoriasis Severity and Psychiatric Outcomes

The PsO severity scores and dermatological life quality index are represented in Table 11, comparing the means between the psychiatric and the non-psychiatric PsO subpopulations.

Spearman’s regression analysis is represented in Supplementary Table S1. Analysis of variance (ANOVA) and Coefficients are calculated for all types of PsO in Table 12, Table 13, Table 14, Table 15, Table 16, Table 17, Table 18, Table 19, Table 20 and Table 21.

## 4. Discussion

This single-center retrospective epidemiological study examined psychiatric comorbidity prevalence and risk factors in 2219 PsO patients from northeastern Romania. To our knowledge, this is the first retrospective, epidemiological study to thoroughly investigate the prevalence of psychiatric comorbidities and their risk factors in PsO patients from Romania. Our core findings revealed that 8% of PsO patients demonstrated at least one psychiatric comorbidity, with depressive disorders comprising the most substantial psychiatric burden (6.6% of the cohort presenting with any form of depression), followed by anxiety-spectrum disorders (2.4% of the cohort). Demographic factors, particularly female gender, younger age, and concurrent psoriatic arthritis, emerged as significant risk factors for elevated psychiatric comorbidity burden.

Understanding the bidirectional relationship between psoriasis and mental health disorders enables physicians to provide holistic care addressing both physical and emotional dimensions, thereby enhancing patient well-being and quality of life. Moreover, a holistic approach is now more important than ever, given the higher pressure modern society places on physical appearance and the overall increased psychological stress levels. The socio-demographic distribution of the actual PsO population is homogeneous and reflects the worldwide data [43].

The three most commonly encountered psychiatric comorbidities are unspecified depressive disorder (120–5.4%), mixed anxiety-depressive disorder (34–1.5%), and mild depression (24–1.1%).

The three most common forms of PsO are PsO vulgaris (1982–89.3%), nail PsO (528–23.5%), and guttate PsO (1070–7.17%), while psoriatic arthritis was encountered in 376 (16.9%) of patients. These findings are in accordance with the general distribution of the different forms of PsO [43].

There are no significant differences regarding the mean and median ages in the psychiatric versus the non-psychiatric PsO subpopulations, proving homogeneity regarding age. Female PsO patients, in comparison to the males, have a 1.37 times higher risk of suffering from significant psycho-emotional stress—RR [95% CI] = 1.37 [0.93–2.01]; however, this result lacks statistical significance (*p* = 0.1). Additionally, female PsO patients are also more prone to develop psychiatric comorbidities—RR [95% CI] = 1.55 [1.12–2.14], *p* = 0.007. In the modern-era general population, apart from schizophrenia, women suffer more from mental health disorders than men, correlating with our findings [44,45].

The age-related patterns observed merit particular attention. Our finding that younger patients (<18 years) demonstrated markedly elevated stress burden (RR 2.032) is consistent with evidence that early-onset psoriasis is associated with heightened psychosocial vulnerability, likely reflecting both increased disease-related distress during developmental periods and reduced coping mechanisms in youth. However, <18 years PsO patients have the lowest incidence of psychiatric comorbidities. Younger people often have more resilience or adaptive capacity to cope with stress, which may delay or reduce the manifestation of clinical psychiatric conditions.

The paradoxical finding that actual psychiatric comorbidity prevalence peaks in the 40–59 age group despite lower stress in this cohort suggests that psychiatric conditions in this group may result from cumulative stress exposure over time rather than acute stress reactivity.

The divergent findings regarding urban versus rural residence are noteworthy. Living in the urban area is associated with a two times higher risk of psycho-emotional stress compared to the rural area—RR [95% CI] 1.305 [1.040–1.637] versus 0.658 [0.472–0.918] (*p* = 0.02). However, living in an urban setting is linked with a lower risk of developing psychiatric diseases, RR [95% CI] 0.51 [0.37–0.69], *p* < 0.001. This suggests that urban healthcare infrastructure, accessibility of mental health services, socioeconomic resources, and social support networks may provide protective factors that mitigate progression from stress to clinical psychiatric disorder. Conversely, rural populations, despite experiencing lower acute stress, may lack equivalent access to preventive mental health services and psychosocial support, potentially explaining their higher psychiatric comorbidity prevalence despite lower baseline stress levels.

Pso patients with PsA have a 54.6% higher risk of associating psychiatric comorbidities, RR [95% CI] = 1.54 [1.06–2.20], *p* < 0.01. Likewise, other studies revealed that PsA patients have a higher risk of mental health disorders, mainly depression and anxiety, compared to Pso patients without PsA, irrespective of Pso severity, and compared to the general population [46].

Female Pso patients have a higher risk than males of developing depression (unspecified depressive disorder RR [95% CI] = 1.87 [1.28–2.73], *p* < 0.01; mild depression RR [95% CI] = 1.39 [0.61–3.16], *p* = 0.43; and moderate depression RR [95% CI] = 2.22 [0.59–8.37], *p* = 0.23), except for the severe form—RR [95% CI] = 0.41 [0.03–4.60], *p* = 0.47. Female gender is also associated with a higher risk of unspecified phobic anxiety disorder, unspecified anxiety disorder, and bipolar disorder, but not statistically significant (*p* > 0.05).

Patients suffering from palmoplantar pustular PsO have the highest risk of suffering from a psychiatric condition (RR [95% CI] = 1.44 [0.87–2.37], *p >* = 0.15), followed by nail PsO (RR [95% CI] = 1.28 [0.93–1.76], *p* = 0.12), PsO vulgaris (RR [95% CI] = 0.97 [0.62–1.51], *p* = 0.35), generalized pustular PsO (RR [95% CI] = 0.95 [0.39–2.31], *p* = 0.91), and guttate PsO (RR [95% CI] = 0.73 [0.39–1.39], *p* = 0.73).

The severity scores of PsO and the impact on quality of life do not correlate positively with the presence of psychiatric comorbidities, except mild PASI, mild PSSI, and DLQI—very large effect (*p* > 0.05). This finding contradicts the common expectation that psychiatric comorbidities, such as anxiety or depression, would exacerbate the severity of PsO due to the interplay between emotional distress and chronic inflammatory conditions. Psycho-emotional stress was not considered a psychiatric comorbidity. Possible explanations for this finding include the anti-inflammatory effect of certain psychotropic medications [47], health behaviors and stress management, surveillance bias (psychiatric patients have more frequent contact with healthcare providers and proactive management of Pso), and subjective disease perception.

The regression analysis shows that schizoaffective disorder-depressive type (t = −2.905; *p* = 0.004) and mild depression (t = −2.296; *p* = 0.022) are negatively associated with PsO vulgaris. Schizoaffective disorder-depressive type (t = 3.549; *p* = 0.000) and psycho-emotional stress (t = 5.195; *p* = 0.000) are positively associated with guttate PsO. Psycho-emotional stress (t = 4.154; *p* = 0.000) and unspecified depression (t = 2.768; *p* = 0.006) are positively associated with palmoplantar pustular PsO. Bipolar disorder (t = 3.103; *p* = 0.002) is positively associated with generalized pustular PsO. Psycho-emotional stress (t = 4.154; *p* = 0.000) and unspecified depression (t = 2.768; *p* = 0.006) are positively associated with palmoplantar pustular PsO. Mild depression (t = 3.307; *p* = 0.001), unspecified anxiety disorder (t = 2.386, *p* = 0.17) and mixed anxiety-depressive disorder (t = 2.030; *p* = 0.042) are positively associated with psoriatic arthritis.

### 4.1. Clinical Implications

Our findings have substantial implications for dermatological practice. First, they underscore the necessity for systematic psychiatric and psychological screening within dermatology clinics. Given the demonstrated elevated psychiatric risk in specific subpopulations (female patients, younger individuals, and those with PsA), targeted screening protocols prioritizing these groups warrant implementation. Second, our findings support the clinical utility of integrating mental health professionals (psychiatrists, psychologists, and licensed clinical counselors) into dermatological care settings, either on-site or via telemedicine consultation.

Practical implementation strategies might include: (a) routine administration of validated screening instruments such as the Patient Health Questionnaire-9 (PHQ-9) for depression and Generalized Anxiety Disorder-7 (GAD-7) for anxiety at dermatology visits, particularly for high-risk subpopulations; (b) clear referral protocols to mental health specialists when screening instruments yield positive results; (c) patient education regarding the bidirectional relationship between stress and psoriasis, potentially through psychoeducational handouts or discussions; and (d) consideration of cognitive-behavioral therapy (CBT) or other psychotherapeutic interventions as adjunctive treatments for PsO patients with concurrent psychiatric conditions

The results of this research could be used for further comparative studies and integrated into more comprehensive national or international databases, which could provide more reliable data.

### 4.2. Study Limitations

The limitations of this retrospective, epidemiological study include the single-center design, with data derived exclusively from one urban academic medical center, which limits generalizability to other geographic regions, healthcare systems, and rural clinical settings; the lack of an attending psychiatrist and/or psychologist to consult the patient during the hospital admission; the retrospective nature of this study, which precluded standardized psychiatric assessment; diagnoses were extracted from medical records using ICD-10 codes rather than validated clinical psychiatric interviews; severity scores were not reported for every patient; inverse and erythrodermic PsO were not documented as a different diagnosis due to local ICD-10 restraints; the dataset did not include documentation regarding whether patients with psychiatric comorbidities received psychiatric medication, preventing evaluation of potential confounding or therapeutic effects of psychopharmacological treatment; the temporal relationship between psychiatric and dermatological diagnoses could not be determined from retrospective records; the small number of cases for certain psychiatric conditions (e.g., only three cases of bipolar disorder and one case of suicidal ideation) limits statistical power for subgroup analyses.

## 5. Conclusions

This retrospective epidemiological study demonstrates that psychiatric comorbidities represent a substantial healthcare burden in PsO patients from northeastern Romania. Depression and anxiety-related disorders emerged as the most prevalent psychiatric conditions, affecting approximately 6.6% and 2.4% of the cohort, respectively. Female patients demonstrated significantly elevated psychiatric comorbidity risk (RR 1.55, *p* = 0.007) compared to males, and younger patients (<18 years) demonstrated substantially elevated stress burden (RR 2.03, *p* = 0.002). Notably, patients with concurrent psoriatic arthritis demonstrated 54.6% elevated psychiatric comorbidity risk (RR 1.546, *p* = 0.002) compared to those without arthritis. These findings underscore the necessity for comprehensive psychiatric screening and integrated mental healthcare within dermatological practice.

Patients aged 60 years and older exhibited lower rates of both psycho-emotional stress and depressive disorders. Living in an urban environment was linked to a higher risk of psycho-emotional stress, whereas patients from rural areas more often met diagnostic criteria for depression and mixed anxiety–depressive disorder. We found no direct correlation between the severity of PsO and its impact on quality of life and the presence of psychiatric comorbidities.

Based on these findings, we recommend the following evidence-based interventions: (1) Routine screening for depression and anxiety using validated instruments (PHQ-9 and GAD-7) should be incorporated as standard practice into all dermatology visits, with particular emphasis on female patients, adolescents, and those with psoriatic arthritis; (2) Clear clinical referral pathways to mental health specialists must be established to ensure appropriate follow-up when screening instruments yield positive results; (3) All dermatology departments should facilitate access to psychiatric or psychological consultation, either through on-site specialist availability or established telemedicine protocols; (4) Patient education regarding the bidirectional stress–psoriasis relationship should be integrated into standard dermatological counseling; and (5) Consideration should be given to psychotherapeutic interventions, particularly cognitive-behavioral therapy, as adjunctive treatment for PsO patients with comorbid psychiatric conditions.

## Figures and Tables

**Table 1:** The main demographics of the PsO study population.

Psoriasis Population (*n* = 2219)	
Gender	
Male	1010 (45.5%)
Female	1209 (54.5%)
Age	
Mean ± SD	52.46 ± 17.77
Age groups	
<18	160 (7.2%)
18–39	296 (13.3%)
40–59	818 (36.9%)
60+	945 (42.6%)
Living setting	
Urban	1173 (52.8%)
Rural	1046 (47.2%)

**Table 2:** Incidence of psychiatric comorbidities and types of psoriasis in the study population (*n* = 2219).

		*n* (%)	*p*-Value
Psychiatric comorbidities	Suicidal ideation	1 (<0.1%)	<0.05
	Unspecified phobic anxiety disorder	7 (0.3%)	
	Unspecified anxiety disorder	19 (0.9%)	
	Mixed anxiety-depressive disorder	34 (1.5%)	
	Bipolar disorder	3 (0.1%)	
	Schizoaffective disorder, depressive type	1 (<0.1%)	
	Unspecified depressive disorder	120 (5.4%)	
	Mild depression	24 (1.1%)	
	Moderate depression	11 (0.5%)	
	Severe depression	3 (0.1%)	
	Paranoid schizophrenia	2 (0.1%)	>0.05
Psoriasis	Psoriasis vulgaris	1982 (89.3%)	<0.05
	Palmo-plantar pustular psoriasis	148 (6.7%)	
	Nail psoriasis	528 (23.8%)	
	Psoriatic arthritis	376 (16.9%)	
	Guttate psoriasis	170 (7.6%)	>0.05
	Generalized pustular psoriasis	66 (3%)	

**Table 3:** The age of PsO patients (*n* = 2219) with and without psychiatric comorbidities.

Age of Psoriasis Patients with	Psychiatric Comorbidities	No Psychiatric Comorbidities
*n*	177	2090
Mean	54.56	52.37
Median	56.00	56.00
Standard deviation	10.751	18.091
Minimum	11	0.33
Maximum	73	90

**Table 4:** Psycho-emotional stress relative risk and 95% confidence intervals, RR [95% CI], in psoriasis patients according to gender, age group, and living setting.

		Psycho-Emotional Stress (*n* = 116)		
		*n*	RR [95% CI]	*p*-Value
Gender	Female	72	1.37 [0.93–2.01]	0.109
	Male	44	0.73 [0.50–1.07]	0.101
Age group	<18	17	2.03 [1.297–3.186]	0.002
	18–39	19	1.22 [0.795–1.897]	0.355
	40–59	52	1.21 [0.935–1.582]	0.145
	60+	28	0.56 [0.394–0.816]	0.002
Living setting	Rural	36	0.65 [0.472–0.918]	0.014
	Urban	80	1.30 [1.040–1.637]	0.022

**Table 5:** Representation of the relative risks and 95% confidence intervals, RR [95% CI], of the psoriasis study population suffering from any psychiatric condition according to gender, living setting, and the presence of psoriatic arthritis.

	Gender				Living Setting				Psoriatic Arthritis			
	*n* (%)		*p*-Value	RR [95% CI]	*n* (%)		*p*-Value	RR [95% CI]	*n* (%)		*p*-Value	RR [95% CI]
	Female	Male			Urban	Rural			**Present**	Absent		
Psoriasis patients with psychiatric comorbidities	115 (5.2%)	62 (2.8%)	0.0074	1.55 [1.12–2.14]	64 (2.9%)	113 (5.1%)	0.0001	0.51 [0.37–0.69]	42 (1.9%)	135 (6.1%)	0.012	1.546 [1.06–2.20] *p* = 0.0024

**Table 6:** Representation of the relative risks and 95% confidence intervals, RR [95% CI], of the psychiatric conditions according to gender and living setting in the psoriasis population of 2219 patients.

	Gender				Living Setting			
	*n* (%)		*p*-Value	RR [95% CI]	*n* (%)		*p*-Value	RR [95% CI]
	Female	Male			Rural	Urban		
Suicidal ideation	1 (<0.1%)	0 (0%)	-	-	1 (<0%)	0 (0%)	-	-
Unspecified phobic anxiety disorder	5 (0.2%)	2 (0.1%)	0.378	2.088 [0.4061–10.7421]	4 (0.2%)	3 (0.1%)	0.528	1.495 [0.3354–6.6654]
Unspecified anxiety disorder	11 (0.5%)	8 (0.4%)	0.300	1.148 [0.4638–2.8448]	13 (0.6%)	6 (0.3%)	0.071	2.429 [0.9268–6.3696]
Mixed anxiety-depressive disorder	16 (0.7%)	17 (0.8%)	0.486	0.786 [0.3993–1.5482]	24 (1.1%)	10 (0.5%)	0.008	2.686 [1.2907–5.5909]
Bipolar disorder	2 (0.1%)	1 (<0.1%)	0.674	1.670 [0.1517–18.4000]	2 (0.1%)	1 (<0.1%)	0.509	2.242 [0.2037–24.6997]
Paranoid schizophrenia	1 (<0.1%)	1 (<0.1%)	0.898	0.835 [0.0523–13.3398]	2 (0.1%)	0 (0%)	-	-
Schizoaffective disorder, depressive type	1 (0%)	0 (0%)	-	-	1 (0%)	0 (0%)	-	-
Unspecified depressive disorder	83 (3.7%)	37 (1.7%)	0.001	1.874 [1.2837–2.7357]	74 (3.3%)	48 (2.1%)	0.002	1.728 [1.2140–2.4621]
Mild depression	15 (0.7%)	9 (0.4%)	0.430	1.392 [0.6119–3.1682]	14 (0.6%)	10 (0.5%)	0.273	1.570 [0.7004–3.5194]
Moderate depression	8 (0.4%)	3 (0.1%)	0.235	2.227 [0.5926–8.3752]	8 (0.4%)	3 (0.1%)	0.105	2.987 [0.7947–11.2321]
Severe depression	1 (<0.1%)	2 (0.1%)	0.475	0.417 [0.0379–4.600]	1 (<0.1%)	2 (0.1%)	0.636	0.560 [0.0509–6.1749]

**Table 7:** Incidence, relative risks, and 95% confidence intervals, RR [95% CI], of different types of psoriasis in the subpopulation suffering from any psychiatric comorbidities.

	Psoriasis Vulgaris			Guttate Psoriasis			Palmo-Plantar Pustular Psoriasis			Generalized Pustular Psoriasis			Nail Psoriasis		
	*n* (%)	*p*-Value	RR [95% CI]	*n* (%)	*p*-Value	RR [95% CI]	*n* (%)	*p*-Value	RR [95% CI]	*n* (%)	*p*-Value	RR [95% CI]	*n* (%)	*p*-Value	RR [95% CI]
Psoriasis patients with psychiatric comorbidities	154 (6.9%)	0.90	0.974 [0.629–1.510]	10 (0.5%)	0.35	0.737 [0.390–1.396]	17 (0,8%)	0.15	1.440 [0.873–2.374]	5 (0.2%)	0.91	0.950 [0.390–2.311]	54 (2.4%)	0.12	1.282 [0.931–1.766]

**Table 8:** The incidence and *p*-values of psychiatric comorbidities in different types of psoriasis. In order to provide an easier reading of this table, *p*-values have been simplified to over 0.05.

	Psoriasis Vulgaris		Guttate Psoriasis		Palmo-Plantar Pustular Psoriasis		Generalized Pustular Psoriasis		Nail Psoriasis		Psoriatic Arthritis	
	*n* (%)	*p*-Value	*n* (%)	*p*-Value	*n* (%)	*p*-Value	*n* (%)	*p*-Value	n(%)	*p*-Value	*n* (%)	*p*-Value
Suicidal ideation	1 (0.05%)	>0.05	0 (0%)	-	0 (0%)	-	0 (0.0%)	-	1 (<0.1%)	0.073	0 (0%)	-
Unspecified phobic anxiety disorder	7 (0.3%)		0 (0%)	-	1 (<0.1%)	> 0.05	0 (0.0%)	-	2 (0.1%)	>0.05	1 (0.05%)	>0.05
Unspecified anxiety disorder	18 (0.8%)		2 (0.1%)	>0.05	0 (0%)	-	1 (0.05%)	>0.05	4 (0.2%)	>0.05	7 (0.3%)	>0.05
Mixed anxiety-depressive disorder	33 (1.5%)		0 (0%)	-	0 (0%)	-	1 (0.05%)	>0.05	6 (0.3%)	>0.05	10 (0.5%)	>0.05
Bipolar disorder	2 (0.1%)		0 (0%)	-	0 (0%)	-	1 (0.05%)	>0.05	0 (0%)	-	1 (0.05%)	>0.05
Paranoid schizophrenia	1 (0.05%)		1 (0.05%)	>0.05	0 (0%)	-	0 (0%)	-	0 (0%)	-	0 (0%)	-
Schizoaffective disorder, depressive type	0 (0%)		1 (0.05%	>0.05	0 (0%)	-	0 (0%)	-	0 (0%)	-	0 (0%)	-
Unspecified depressive disorder	100 (4.5%)		7 (0,3%)	>0.05	16 (0.7%)	>0.05	3 (0.1%)	>0.05	44 (2%)	>0.05	26 (1.2%)	>0.05
Mild depression	18 (0.8%)		1 (0.05%)	>0.05	2 (0.1%)	>0.05	1 (0.0%)	>0.05	2 (0.1%)	>0.05	10 (0.5%)	>0.05
Moderate depression	10 (0.5%)		1 (0.0%)	>0.05	1 (<0.1%)	>0.05	0 (0.0%)	-	4 (0.2%)	>0.05	4 (0.2%)	>0.05
Severe depression	3 (0.1%)		0 (0%)	-	0 (0%)	-	0 (0%)	-	0 (0%)	-	1 (0.05%)	>0.05

**Table 9:** The distribution of psychiatric comorbidities in psoriasis patients by age groups and the associated relative risks and 95% confidence intervals, RR [95% CI].

	Age Group								
	<18	RR [95% CI]	18–39	RR [95% CI]	40–59	RR [95% CI]	60+	RR [95% CI]	*p*-Value
Psoriasis patients with psychiatric comorbidities	2 (0.1%)	0.157 [0.039–0.632]	10 (0.5%)	0.424 [0.225–0.796]	105 (4.7%)	1.609 [1.311–1.975]	60 (2.7%)	0.796 [0.613–1.034]	<0.05

**Table 10:** The distribution of psychiatric comorbidities in detail in the psoriasis study population by age groups.

Age Group	<18		18–39		40–59		60+	
	*n* (%)	RR [95% CI]	*n* (%)	RR [95% CI]	*n* (%)	RR [95% CI]	*n* (%)	RR [95% CI]
Psycho-emotional stress	17 (10.6%)	2.032 [1.229–3.361] *p* = 0.006	19 (6.4%)	1.228 [0.771–1.954] *p* = 0.387	52 (6.4%)	1.216 [0.918–1.610] *p* = 0.172	28 (3%)	0.567 [0.389–0.826] *p* = 0.003
Suicidal ideation	0	-	0	-	1 (0.1%)	2.713 [0.382–19.282] *p* = 0.319	0	-
Unspecified phobic anxiety disorder	0	-	1 (0.3%)	1.071 [0.150–7.628 *p* = 0.945	3 (0.4%)	1.163 [0.374–3.612] *p* = 0.795	3 (0.3%)	1.006 [0.324–3.126] *p* = 0.991
Unspecified anxiety disorder	2 (1.3%)	1.460 [0.362–5.889] *p* = 0.595	0	-	13 (1.6%)	1.856 [1.073–3.211] *p* = 0.027	4 (0.4%)	0.494 [0.185–1.320] *p* = 0.160
Mixed anxiety-depressive disorder	0	-	1 (0.3%)	0.220 [0.031–1.571] *p* = 0.131	16 (2%)	1.277 [0.778–2.094] *p* = 0.333	17 (1.8%)	1.174 [0.727–1.897] *p* = 0.512
Bipolar disorder	0	-	0	-	2 (0.2%)	1.808 [0.452–7.244] *p* = 0.403	1 (0.1%)	0.783 [0.110–5.563] *p* = 0.807
Paranoid schizophrenia	0	-	1 (0.3%)	3.748 [0.526–26.699] *p* = 0.187	1 (0.1%)	1.356 [0.191–9.641] *p* = 0.761	0	-
Schizoaffective disorder, depressive type	0	-	0	-	1 (0.1%)	2.713 [0.382–19.282] *p* = 0.319	0	-
Unspecified depressive disorder	0	-	7 (2.4%)	0.437 [0.207–0.926] *p* = 0.031	76 (9.3%)	1.718 [1.357–2.175] *p* < 0.0001	37 (3.9%)	0.724 [0.521–1.006] *p* = 0.054
Mild depression	0	-	0	-	16 (2%)	1.808 [1.103–2.966] *p* = 0.019	8 (0.8%)	0.783 [0.390–1.570] *p* = 0.490
Moderate depression	0	-	1 (0.3%)	0.682 [0.096–4.854] *p* = 0.702	7 (0.9%)	1.726 [0.820–3.633] *p* = 0.150	3 (0.3%)	0.640 [0.206–1.989] *p* = 0.441
Severe depression	0	-	0	-	3 (0.4%)	2.713 [0.873–8.429] *p* = 0.084	0	-

**Table 11:** Severity scores of psoriasis and the impact on quality of life according to the presence or absence of psychiatric comorbidities.

	Severity	Psychiatric Comorbidities	No Psychiatric Comorbidities	*p*-Value
		Mean		
PASI (*n* = 449)	Mild	2.567	2.249	0.139
	Moderate	7.8	7.993	0.530
	Severe	19.823	21.844	0.021
PSSI (*n* = 10)	Mild	5	2	0.157
	Moderate	-	6	-
	Severe	12	38.80	0.199
NAPSI (*n* = 12)		14	46.80	0.139
DLQI (*n* = 274)	No effect on patient’s life	-	0.259	-
	Small effect	3.0	3.542	0.695
	Moderate effect	8.5	8.667	0.839
	Very Large effect	17.667	15.909	0.379
	Extremely large effect	22.60	24.889	0.525

PASI = Psoriasis Area Severity Index; PSSI = Psoriasis Scalp Severity Index; NAPSI = Nail Psoriasis Severity Index; DLQI = Dermatological Life Quality Index.

**Table 12:** Analysis of variance (ANOVA) for psoriasis vulgaris.

ANOVA ^a^						
Model		Sum of Squares	df	Mean Square	F	Sig.
1	Regression	1.298	2	0.649	6.838	0.001 ^b^
	Residual	210.389	2216	0.095		
	Total	211.687	2218			

^a^ Dependent variable: psoriasis vulgaris. ^b^ Predictors: (constant), mild depression, and schizoaffective disorder—depressive type.

**Table 13:** Coefficient analysis for psoriasis vulgaris.

Coefficients ^a^						
Model		Unstandardized Coefficients		Standardized Coefficients	t	Sig.
		B	Std. Error	**Beta**		
1	(Constant)	0.895	0.007		136.081	0.000
	Schizoaffective disorder depressive type	−0.895	0.308	−0.062	−2.905	0.004
	Mild depression	−0.145	0.063	−0.049	−2.296	0.022

^a^ Dependent variable: PsO vulgaris.

**Table 14:** Analysis of variance (ANOVA) for guttate psoriasis.

ANOVA ^a^						
Model		Sum of Squares	df	Mean Square	F	Sig.
1	Regression	7.010	3	2.337	34.512	0.000 ^b^
	Residual	149.966	2215	0.068		
	Total	156.976	2218			

^a^ Dependent variable: guttate psoriasis. ^b^ Predictors: (constant), schizoaffective disorder depressive type, and psycho-emotional stress.

**Table 15:** Coefficient analysis for guttate psoriasis.

Coefficients ^a^						
Model		Unstandardized Coefficients		Standardized Coefficients	t	Sig.
		B	Std. Error	**Beta**		
1	(Constant)	0.213	0.020		10569	0.000
	Schizoaffective disorder depressive type	0.924	0.260	0.074	3.549	0.000
	Psycho-emotional stress	0.129	0.025	0.108	5.195	0.000

^a^ Dependent Variable: Guttate psoriasis.

**Table 16:** Analysis of variance (ANOVA) for palmoplantar pustular psoriasis.

ANOVA ^a^						
Model		Sum of Squares	df	Mean Square	F	Sig.
1	Regression	1.626	2	0.813	13.199	0.000 ^b^
	Residual	136.503	2216	0.062		
	Total	138.129	2218			

^a^ Dependent variable: palmo-plantar pustular psoriasis. ^b^ Predictors: (constant), unspecified depression and psycho-emotional stress.

**Table 17:** Coefficients analysis for palmoplantar pustular psoriasis.

Coefficients ^a^						
Model		Unstandardized Coefficients		Standardized Coefficients	t	Sig.
		B	Std. Error	Beta		
1	(Constant)	0.058	0.006		10.477	0.000
	Psycho-emotional stress	0.098	0.024	0.088	4.154	0.000
	Unspecified depression	0.065	0.023	0.059	2.768	0.006

^a^ Dependent variable: palmo-plantar pustular psoriasis.

**Table 18:** Analysis of variance (ANOVA) for generalized pustular psoriasis.

ANOVA ^a^						
Model		Sum of Squares	df	Mean Square	F	Sig.
1	Regression	0.277	1	0.277	9.627	0.002 ^b^
	Residual	63.760	2217	0.029		
	Total	64.037	2218			

^a^ Dependent variable: generalized pustular psoriasis. ^b^ Predictors: (constant), bipolar disorder.

**Table 19:** Coefficient analysis for generalized pustular psoriasis.

Coefficients ^a^						
Model		Unstandardized Coefficients		Standardized Coefficients	t	Sig.
		B	Std. Error	Beta		
1	(Constant)	0.029	0.004		8.142	0.000
	Bipolar disorder	0.304	0.098	0.066	3.103	0.002

^a^ Dependent variable: generalized pustular psoriasis.

**Table 20:** Analysis of variance (ANOVA) for psoriatic arthritis.

ANOVA ^a^						
Model		Sum of Squares	df	Mean Square	F	Sig.
1	Regression	2.838	3	0.946	6.772	0.000 ^b^
	Residual	309.450	2215	0.140		
	Total	312.288	2218			

^a^ Dependent variable: psoriatic arthritis. ^b^ Predictors: (constant), mild depression, unspecified anxiety disorder, and mixed anxiety-depressive disorder.

**Table 21:** Coefficient analysis for psoriatic arthritis.

Coefficients ^a^						
Model		Unstandardized Coefficients		Standardized Coefficients	t	Sig.
		B	Std. Error	Beta		
1	(Constant)	0.163	0.008		20.175	0.000
	Unspecified anxiety disorder	0.205	0.086	0.050	2.386	0.017
	Mixed anxiety-depressive disorder	0.131	0.065	0.043	2.030	0.042
	Mild depression	0.254	0.077	0.070	3.307	0.001

^a^ Dependent variable: psoriatic arthritis.

## Data Availability

The original contributions presented in this study are included in the article and Supplementary Material. Further inquiries can be directed to the corresponding authors.

## Supplementary Materials

The following supporting information can be downloaded at: https://www.mdpi.com/article/10.3390/medicina61122190/s1, Table S1: Spearman’s regression analysis.
