# Supplementary material for: Psychiatric Comorbidities in Patients with Psoriasis: A 10-Year Retrospective Epidemiological Study from a Tertiary University Center in Northeastern Romania
Source: Medicina (Kaunas). 2025 Dec 10;61(12):2190. doi: 10.3390/medicina61122190 (PMC12735106; doi:10.3390/medicina61122190)
Supplement: Supplementary file 1 [file medicina-61-02190-s001.zip › medicina-4031168-supplementary.pdf]

			Psoriasis vulgaris	Guttate psoriasis	Psychiatric comorbidities	Paranoid schizofrenia	Generalised pustular psoriasis	Bipolar disorder	Moderate Depression	Schizoaffective disorder depressive type	Mixed anxious-depressive disorder	Nail psoriasis	Psoriatic arthritis	Unspecified phobic anxiety disorder	Mild Depression	Unspecified Depression	Unspecified anxiety disorder	Suicidal ideation	Psycho-emotional stress	Palmoplantar pustular psoriasis
**Spearman's rho**	**Psoriasis vulgaris**	Correlation Coefficient	1,000	-,213**	-0,022	-0,038	-,231**	-0,027	0,004	-,061**	0,031	,087**	,063**	0,019	-,048*	-,046*	0,016	0,007	0,003	-,434**
		Sig. (2-tailed)		0,000	0,299	0,072	0,000	0,204	0,864	0,004	0,141	0,000	0,003	0,360	0,022	0,029	0,443	0,730	0,904	0,000
	**Guttate psoriasis**	Correlation Coefficient	-,213**	1,000	-0,022	,048*	-0,021	-0,011	0,004	,074**	-0,036	-,046*	-,044*	-0,016	-0,014	-0,016	0,010	-0,006	,123**	-0,036
		Sig. (2-tailed)	0,000		0,294	0,024	0,334	0,618	0,858	0,001	0,091	0,032	0,037	0,446	0,518	0,439	0,637	0,773	0,000	0,088
	**Psychiatric comorbidities**	Correlation Coefficient	-0,022	-0,022	1,000	,102**	-0,003	,125**	,240**	,072**	,424**	,046*	,053*	,191**	,355**	,812**	,316**	,072**	,058**	0,035
		Sig. (2-tailed)	0,299	0,294		0,000	0,903	0,000	0,000	0,001	0,000	0,029	0,012	0,000	0,000	0,000	0,000	0,001	0,006	0,103
	**Paranoid schizofrenia**	Correlation Coefficient	-0,038	,048*	,102**	1,000	-0,005	-0,001	-0,002	,707**	-0,004	-0,017	-0,014	-0,002	-0,003	-0,007	-0,003	-0,001	-0,007	-0,008
		Sig. (2-tailed)	0,072	0,024	0,000		0,804	0,959	0,920	0,000	0,860	0,429	0,523	0,937	0,882	0,735	0,895	0,976	0,740	0,705
	**Generalised pustular psoriasis**	Correlation Coefficient	-,231**	-0,021	-0,003	-0,005	1,000	,066**	-0,012	-0,004	0,000	0,014	,105**	-0,010	0,007	-0,007	0,013	-0,004	0,018	-0,036
		Sig. (2-tailed)	0,000	0,334	0,903	0,804		0,002	0,561	0,861	0,991	0,501	0,000	0,643	0,730	0,753	0,556	0,861	0,384	0,088
	**Bipolar disorder**	Correlation Coefficient	-0,027	-0,011	,125**	-0,001	,066**	1,000	-0,003	-0,001	-0,005	-0,021	0,016	-0,002	-0,004	,100**	-0,003	-0,001	-0,009	-0,010
		Sig. (2-tailed)	0,204	0,618	0,000	0,959	0,002		0,903	0,971	0,829	0,333	0,449	0,922	0,856	0,000	0,872	0,971	0,684	0,643
	**Moderate Depression**	Correlation Coefficient	0,004	0,004	,240**	-0,002	-0,012	-0,003	1,000	-0,001	-0,009	0,021	0,037	-0,004	-0,007	,295**	-0,007	-0,001	0,012	0,007
		Sig. (2-tailed)	0,864	0,858	0,000	0,920	0,561	0,903		0,944	0,678	0,327	0,085	0,852	0,728	0,000	0,757	0,944	0,564	0,747
	**Schizoaffective disorder depressive type**	Correlation Coefficient	-,061**	,074**	,072**	,707**	-0,004	-0,001	-0,001	1,000	-0,003	-0,012	-0,010	-0,001	-0,002	-0,005	-0,002	0,000	-0,005	-0,006
		Sig. (2-tailed)	0,004	0,001	0,001	0,000	0,861	0,971	0,944		0,901	0,576	0,652	0,955	0,917	0,811	0,926	0,983	0,814	0,789
	**Mixed anxious-depressive disorder**	Correlation Coefficient	0,031	-0,036	,424**	-0,004	0,000	-0,005	-0,009	-0,003	1,000	-0,018	0,041	-0,007	-0,013	0,035	-0,012	-0,003	-0,029	-0,033
		Sig. (2-tailed)	0,141	0,091	0,000	0,860	0,991	0,829	0,678	0,901		0,396	0,051	0,741	0,539	0,099	0,585	0,901	0,168	0,116
	**Nail psoriasis**	Correlation Coefficient	,087**	-,046*	,046*	-0,017	0,014	-0,021	0,021	-0,012	-0,018	1,000	,097**	0,006	-0,038	,072**	-0,006	0,038	,049*	-0,014
		Sig. (2-tailed)	0,000	0,032	0,029	0,429	0,501	0,333	0,327	0,576	0,396		0,000	0,766	0,074	0,001	0,778	0,074	0,020	0,521
		N	2219	2219	2219	2219	2219	2219	2219	2219	2219	2219	2219	2219	2219	2219	2219	2219	2219	2219
	**Psoriatic arthritis**	Correlation Coefficient	,063**	-,044*	,053*	-0,014	,105**	0,016	0,037	-0,010	0,041	,097**	1,000	-0,004	,069**	0,030	,049*	-0,010	-0,004	-,077**

		Sig. (2-tailed)	0,003	0,037	0,012	0,523	0,000	0,449	0,085	0,652	0,051	0,000		0,851	0,001	0,156	0,020	0,652	0,868	0,000
	**Unspecified phobic anxiety disorder**	Correlation Coefficient	0,019	-0,016	,191**	-0,002	-0,010	-0,002	-0,004	-0,001	-0,007	0,006	-0,004	1,000	-0,006	-0,013	-0,005	-0,001	0,023	0,017
		Sig. (2-tailed)	0,360	0,446	0,000	0,937	0,643	0,922	0,852	0,955	0,741	0,766	0,851		0,782	0,527	0,806	0,955	0,281	0,419
	**Mild Depression**	Correlation Coefficient	-,048*	-0,014	,355**	-0,003	0,007	-0,004	-0,007	-0,002	-0,013	-0,038	,069**	-0,006	1,000	,437**	-0,010	-0,002	-0,025	0,007
		Sig. (2-tailed)	0,022	0,518	0,000	0,882	0,730	0,856	0,728	0,917	0,539	0,074	0,001	0,782		0,000	0,647	0,917	0,248	0,743
	**Unspecified Depression**	Correlation Coefficient	-,046*	-0,016	,812**	-0,007	-0,007	,100**	,295**	-0,005	0,035	,072**	0,030	-0,013	,437**	1,000	0,021	,089**	,060**	,064**
		Sig. (2-tailed)	0,029	0,439	0,000	0,735	0,753	0,000	0,000	0,811	0,099	0,001	0,156	0,527	0,000		0,322	0,000	0,005	0,003
	**Unspecified anxiety disorder**	Correlation Coefficient	0,016	0,010	,316**	-0,003	0,013	-0,003	-0,007	-0,002	-0,012	-0,006	,049*	-0,005	-0,010	0,021	1,000	-0,002	,044*	-0,025
		Sig. (2-tailed)	0,443	0,637	0,000	0,895	0,556	0,872	0,757	0,926	0,585	0,778	0,020	0,806	0,647	0,322		0,926	0,038	0,242
	**Suicidal ideation**	Correlation Coefficient	0,007	-0,006	,072**	-0,001	-0,004	-0,001	-0,001	0,000	-0,003	0,038	-0,010	-0,001	-0,002	,089**	-0,002	1,000	-0,005	-0,006
		Sig. (2-tailed)	0,730	0,773	0,001	0,976	0,861	0,971	0,944	0,983	0,901	0,074	0,652	0,955	0,917	0,000	0,926		0,814	0,789
	**Psycho-emotional stress**	Correlation Coefficient	0,003	,123**	,058**	-0,007	0,018	-0,009	0,012	-0,005	-0,029	,049*	-0,004	0,023	-0,025	,060**	,044*	-0,005	1,000	,091**
		Sig. (2-tailed)	0,904	0,000	0,006	0,740	0,384	0,684	0,564	0,814	0,168	0,020	0,868	0,281	0,248	0,005	0,038	0,814		0,000
	**Palmoplantar pustular psoriasis**	Correlation Coefficient	-,434**	-0,036	0,035	-0,008	-0,036	-0,010	0,007	-0,006	-0,033	-0,014	-,077**	0,017	0,007	,064**	-0,025	-0,006	,091**	1,000
		Sig. (2-tailed)	0,000	0,088	0,103	0,705	0,088	0,643	0,747	0,789	0,116	0,521	0,000	0,419	0,743	0,003	0,242	0,789	0,000	

****. Correlation is significant at the 0.01 level (2-tailed).**

***. Correlation is significant at the 0.05 level (2-tailed).**

Table S1. Spearman’s regression analysis.
